# Correlates of Rehabilitation Length of Stay in Asian Traumatic Brain Injury Inpatients in a Superaged Country: A Retrospective Cohort Study

**DOI:** 10.3390/life15071136

**Published:** 2025-07-18

**Authors:** Karen Sui Geok Chua, Zachary Jieyi Cheong, Emily Yee, Rathi Ratha Krishnan

**Affiliations:** 1 Institute of Rehabilitation Excellence (IREx), Tan Tock Seng Hospital, Singapore 308433, Singapore; emily_yee@ttsh.com.sg (E.Y.); rathi_ratha_krishnan@ttsh.com.sg (R.R.K.); 2 Lee Kong Chian School of Medicine, Nanyang Technological University, Singapore 308232, Singapore; zcheong006@e.ntu.edu.sg; 3 Yong Loo Lin School of Medicine, National University of Singapore, Singapore 117597, Singapore; 4 Rehabilitation Research Institute of Singapore (RRIS), Nanyang Technological University, Singapore 308232, Singapore; 5 Clinical Research and Innovation Office (CRIO), Tan Tock Seng Hospital, Singapore 308433, Singapore

**Keywords:** traumatic brain injury, head injury, neurotrauma, rehabilitation, length of stay, post-traumatic amnesia, functional Independence measure, neurosurgery, discharge

## Abstract

Background: While Asia contributes 44.3% of traumatic brain injuries (TBI) worldwide, data regarding Asian TBI inpatient rehabilitation length of stay (RLOS) is scarce. A retrospective cohort study was conducted to determine correlates of inpatient RLOS (days) and prolonged RLOS >30 days (PRLOS > 30). (2) Methods: Data extraction of discharged inpatient records was performed from 2018 to 2024. Dependent variables included RLOS (days) and PRLOS > 30. Independent variables included demographic characteristics, TBI severity (emergency-room Glasgow Coma Scale-GCS), admission/discharge Functional Independence Measure (FIM), intra-rehabilitation complications, post-traumatic amnesia (PTA) duration, and discharge placement. (3) Results: Altogether, 289 data sets were analysed, median (IQR) age, 64 (28) years, 78.9% (228/289) males, and 79.6% (230/289) Chinese. Median (IQR) RLOS was 28 (21) days, with PRLOS >30 at 39.8% (115/289); RLOS of 44 (19.5) days. PRLOS > 30 was significantly associated with PTA duration >28 days (OR 4.01, 95% CI 1.90–8.45, *p* < 0.001), admission FIM ≤ 40/126 (OR 4.71, 95% CI 2.32–9.59, *p* < 0.001), delayed neurosurgical complications (OR 4.74, 95% CI 1.28–17.6, *p* = 0.02) and discharge to non-home destination (OR 2.75. 95% CI 1.12–6.76, *p* = 0.03). (4) Conclusion: PRLOS >30 was significantly associated with longer PTA > 4 weeks, lower admission FIM score, delayed neurosurgical complications, and discharge to a nursing home.

## 1. Introduction

Traumatic Brain Injury (TBI) is a major cause of mortality and morbidity and is regarded as a major public health condition with Years Lived with Disability (YLDs) estimated at 8.1 million [1,2]. From 1990 to 2016, an 8.4% increase in the world’s age-standardized prevalence rates of TBI was reported, in association with a 3.6% increase in the age-standardised incidence rates of TBIs [2]. Within the United States, ~214,110 TBI-related hospitalisations and~ 69,473 TBI-related deaths were reported in 2020 and 2021, respectively, while ~3.2- 5.3 million Americans live with a TBI-related disability [1,2]. Falls and motor vehicle accidents are the commonest causes of TBI, and world population aging is estimated to fuel further rises in TBI incidence [2].

South Asia and East Asia are the 2 world regions with the highest number of TBI patients, accounting for 44.3% of global cases [3]. In Singapore, trauma constitutes the 5th principal cause of death. In tandem with global population aging and increasing age of TBI onset, falls have overtaken road traffic accidents as the predominant cause of TBI-related hospitalisation [4,5].

Regarding moderate to severe (ms-TBI), inpatient, physician-led, multidisciplinary, goal-directed rehabilitation programme are evidence-based interventions to reduce mortality, complications, disability, improve function, quality of life, and reduce acute hospital length of stay [1,5,6]. Current literature suggests that early transfers to inpatient TBI rehabilitation, particularly within 6 weeks of TBI, reduce total healthcare length of stay [7,8,9,10].

A US study that compared TBI between Caucasians and Asians highlighted notable differences in TBI incidence, severity, access to TBI rehabilitation services, and functional outcomes. In this study, Kuerban et al. reported that Asian TBI survivors tended to have milder TBI severity, but failed to make the same improvements as Hispanics and Caucasians at rehabilitation discharge and 1-year follow-up [11]. This was particularly so in foreign-born Asians compared to their native-born counterparts, indicating that nativity may play a role in recovery extent and future trajectory. Furthermore, Brenner et al. reported that amongst TBI in a US trauma database, Asians and people of colour also faced disparities in access to post-acute further care with lower rates of discharge to rehabilitation, indicating systemic differences in accessing TBI rehabilitation [12].

Duration of days spent during inpatient rehabilitation, termed rehabilitation length of stay (RLOS), is a major contributor to direct billed rehabilitation costs [6,13]. In comparison to Asia’s substantial contributions to the global TBI disease burden, data from Southeast Asia TBI rehabilitation units are sparse [3]. In Singapore, 2 studies, a decade apart, reported public healthcare expenditure outcomes. A study of adult tertiary inpatient rehabilitation in 2010 estimated a median billed cost of S$7845.50 (range: S$970.55–$44,817.20) per TBI inpatient episode, 72% of which were costs related to bed and board charges, nursing, occupational therapy, and physiotherapy services [13]. RLOS (mean 29.9 days) and changes in function were the only 2 factors that significantly impacted inpatient costs [13]. Subsequently, a 2021 study of paediatric TBI in Singapore’s largest public healthcare institution for children, reported a median RLOS of 13.5 days and a median billed cost of S$8361.00 (IQR: S$3543–S$25,232), 40% of which were costs contributed by daily ward bed costs and another 17% contributed from rehabilitation services [14]. Thus, RLOS has a direct influence on inpatient rehabilitation billed costs, with potential implications on the overall healthcare burden of TBI.

The aims of this study were to (i) determine the RLOS (days), prevalence, and PRLOS > 30 in a cohort of South-east Asian TBI during inpatient rehabilitation, (ii) determine correlates of PRLOS > 30 with regard to demographic and TBI acute factors, intra-rehabilitation variables, discharge functional outcomes, and discharge destination.

## 2. Materials and Methods

### 2.1. Study Design and Setting

A retrospective study of discharged electronic medical records (EMRs) from a single public healthcare institution’s tertiary rehabilitation centre (Tan Tock Seng Hospital (TTSH) Rehabilitation Centre’s brain injury rehabilitation unit) was conducted from 1 November 2018 and 1 March 2024. This unit has direct links to a level II trauma centre at TTSH and the National Neuroscience Institute’s acute neurotrauma service.

### 2.2. Ethics Statement

The study obtained institutional review board ethics approval from the National Healthcare Group Domain Specific Review Board. (NHG DSRB 2023/00873, date of approval 9 May 2024, approver Dr Koh Kwong Fah). The study was registered with www.clinicaltrials.gov (NCT 06704997). All research was completed according to the Declaration of Helsinki. A waiver of consent was obtained as patients had been discharged or lost to follow-up after their initial inpatient rehabilitation episode, rendering retrospective consent laborious. (refer Supplementary Material S1; for ethics approval letter, and 2; list of variables) Data obtained from electronic medical records were de-identified after extraction and after the use of patient identifiers to access electronic discharge hospital records.

### 2.3. Study Participants

The study’s data inclusion criteria were (1) first -ever TBI confirmed by neurosurgeons with neuroimaging (CT or MRI brain), (2) age at least 21 years at TBI onset, (3) admitted to inpatient rehabilitation unit within 3 months of TBI from acute neurotrauma units, and (4) completed inpatient rehabilitation with goal attainment and a physicians’ discharge plan. Patients who were discharged against medical advice before completion of rehabilitation were excluded.

### 2.4. Description of Rehabilitation Programme

Tan Tock Seng Hospital (TTSH) Rehabilitation Centre is a 95-bedded inpatient tertiary hospital with direct links to a level II trauma centre at the TTSH campus. TTSH is the largest and oldest public hospital of the National Healthcare Group (NHG), Singapore, and serves a catchment of 1.5 million residents in Central and Northern Singapore. All inpatients admitted to the centre are screened through physiatry consultations or staff specialist rounds for rehabilitation indications and medical stability prior to transfer to the TTSH brain injury rehabilitation unit. During the study duration, the TBI rehabilitation unit was staffed at 17–20 beds with a ratio of 1 therapist/nurse to 7–8 inpatients, including physiotherapists, occupational therapists, speech and language pathologists, psychologists, and medical social workers. Weekly dietetics consultations were also provided.

The TBI rehabilitation programme involves inter-disciplinary rehabilitation therapies (3 h daily, 5.5 days a week) led by a rehabilitation physician consultant, with team goal setting, weekly staff multi-disciplinary meetings, and estimated discharge dates from the TBI rehabilitation unit. These were planned in accordance with the attainment of team goals and the family’s care plan or discharge destination. During inpatient rehabilitation, functional goals, estimated RLOS (days), and post-discharge destination were determined without influence of insurance payors’ stipulations on RLOS, functional status, or inpatient bill size, but in accordance with multidisciplinary team goal setting and attainment in tandem with discharge planning. For inpatients whose primary discharge destination was a nursing home or those who were awaiting the arrival of paid caregivers in the form of foreign domestic workers, they were transferred to interim care facilities, once inpatient rehabilitation goals were achieved or a functional plateau.

The main rehabilitation outcome measure used was the Functional Independence Measure^®^ [15]. (18 minimum score–126 maximum score), measured within 72 h of rehabilitation admission (Ta-FIM) and planned rehabilitation discharge (Td-FIM). Hence, FIM ≥ 91 indicated modified-to-independent levels, implying a minimum of 6–7 points each, aggregated across the 18 domains [16].

Since 1 April 1984, all Singapore citizens, permanent residents, and resident foreigners’ hospitalisation, day surgery, and partial outpatient costs are covered by Medisave, Singapore’s national medical scheme, which is contributed to from an individual’s income [17].

### 2.5. Study Variables

Electronic medical records (EMRs) were identified through the bed admissions management unit and functional data from a rehabilitation standing database. Data extraction using discharged EMRs was performed independently by a medical student (ZJYC) using CDOC (prior to September 2022) and Epic Systems (from September 2022), and data was used to construct a case record form consisting of independent variables described below, and FIM data. The primary dependent variables were: (i) RLOS (days), calculated as the number of days between the dates of rehabilitation admission and discharge inclusive, as obtained from the first page of individual patients’ electronic discharge summaries; and (ii) RLOS > 30 days was classified as a prolonged RLOS (PRLOS > 30). While the definition of what constitutes an extended or prolonged rehabilitation stay can vary between studies, where some authors may refer to the 75th–95th percentile of RLOS as being prolonged, we determined RLOS >30 days to be prolonged RLOS (PRLOS > 30). Historically, 30 days was the center’s recommended standard duration of inpatient RLOS for TBI inpatient admissions [13,18,19,20].

Acute LOS (ALOS) was calculated as the number of days between acute hospital admission and admission to rehabilitation, inclusive. For patients with acute unplanned return (ACUR) to acute TTSH units during their inpatient rehabilitation stay, durations (days) in acute units were subtracted from the total rehabilitation days (RLOS) to obtain the individual RLOS (days).

Independent variables extracted for analysis were categorised as:

(i) Demographic variables:

Age in years, sex (male/female), race (Chinese/non-Chinese), presence of premorbid employment.

(ii) Pre-admission comorbidities:

Stroke, TBI, hypertension, hyperlipidemia, diabetes mellitus, ischemic heart disease, end-stage renal failure (ESRF), end-stage liver failure (ESLF), chronic epilepsy, chronic smoking, chronic alcohol consumption, known psychiatric disorder, cancer, or dementia. These comorbidities and individual age at TBI onset were used to derive the age-adjusted Charlson Comorbidity Index (AACCI) score [21]. This was subsequently re-classified into low (AACCI 0–2) and high (AACCI ≥ 3) groups for analysis.

(iii) Acute TBI characteristics:

Mechanism of TBI -motor vehicle accident [MVA], falls, sports, admission Glasgow Coma Scale (GCS), acute length of stay (ALOS) (days).

(iv) Acute TBI management:

Intensive care unit (ICU) stay (days) and neurosurgical interventions (external ventricular drainage, craniotomy, decompressive craniectomy, clot evacuation, and ventriculoperitoneal shunting), and presence of tracheostomy [22].

(v) Intra-rehabilitation characteristics:

RLOS (days), presence of motor impairment, and duration of posttraumatic amnesia (PTA).

PTA was defined as the time (days) from TBI till the return of continuous memory of ongoing events. PTA duration was measured by rehabilitation professionals using either the Westmead PTA scale (WPTAS) or Orientation-Log (O-Log) [23,24,25]. For patients < 80 years of age without prior causes of cognitive impairment (dementia, stroke, previous TBI), WPTAS was used to assess PTA duration in days. WPTAS consists of 7 orientation items and 5 memory items assessing prospective memory. The scale is administered daily, and PTA emergence levels are achieved on the first of 3 days upon obtaining a score of 12/12 or until the day of discharge from inpatient rehabilitation if 12/12 is not achieved by then. If the patient failed to achieve **a** criterion score on either WMPTAS or O-Log by discharge, patients were deemed PTA-non-emerged [23,24]. The O-Log, rather than WPTAS, was used if patients were >80 years of age or had prior stroke, dementia, or known cognitive impairment [25].

(vi) Intra-rehabilitation complications:

Defined as unexpected new intra-rehabilitation problems that required treatment and/or disrupted rehabilitation after rehabilitation admission.

Classified as (i) medical (e.g., infections -nosocomial infections, urinary tract infections, pneumonia), venous thromboembolism, gastrointestinal complications); or (ii) neurosurgical complications (e.g., post-traumatic seizures, new intracranial collections or infections, hydrocephalus, syndrome of the trephined brain)

The number of ACUR was recorded if transfer out to higher levels of care was needed due to medical decompensation [26].

(vii) Functional status during rehabilitation

Total FIM^®^ score (18–126) recorded within 72 h of admission and discharge by certified therapists [15,16]. These were categorised as total admission FIM (Ta-FIM), total discharge FIM (Td-FIM), admission motor-FIM, admission cognitive-FIM, discharge motor-FIM, and discharge cognitive-FIM.

Discharge destination was categorised as: home, and others (nursing home or community hospital, or death).

### 2.6. Statistical Analysis

Data was collected using patient identifiers, and a standardised case record form was built, then de-identified by an independent medical student (ZJYC). The data was then sequentially processed through NHG-REDcap, then Microsoft Excel, and analyzed using IBM SPSS v29 and R v4.4.1. Non-normally distributed continuous variables (Kolmogorov-Smirnov test, *p* < 0.05) were presented as median and interquartile range, while nominal data were presented as numbers with percentages. Non-parametric tests (Mann-Whitney U and Kruskal-Wallis) were used for group comparisons. For predicting PRLOS > 30, variables with *p* < 0.05 in univariate analyses were identified as potential predictors. After collinearity assessment using Chi-square, Spearman’s, and point-biserial correlations, we built the binary logistic and multilinear regression models.

Following these analyses, binary logistic regression and multilinear regression were performed. The final model incorporated 8 variables: AA-CCI (age-adjusted Charlson Comorbidity Index), ICU admission, presence of Motor Impairment, PTA Duration > 28 Days, (PTA > 28 days), Ta-FIM, intra-rehabilitation medical complications, intra-rehabilitation neurosurgical complications, and discharge destination. PTA > 28 days was used as a predictor, as it classified TBI as being very severe. Subsequently, the final multilinear regression model incorporated 10 variables: employment status before TBI, ICU duration, ALOS, presence of motor impairment, PTA duration, Ta-motor FIM, Ta-cognitive FIM, intra-rehabilitation medical complications, intra-rehabilitation neurosurgical complications, and discharge destination.

After collinearity assessment, AA-CCI was selected over individual comorbidities like hyperlipidemia as it provided a more comprehensive measure of comorbidities. Additionally, chi-square testing showed a significant association between hyperlipidemia and AA-CCI (χ^2^ = 69.44, *p* < 0.001), suggesting potential collinearity if both were included. Similarly, significant collinearity was observed between ACUR and intra-rehabilitation neurosurgical complications (χ^2^ = 41.63, *p* < 0.001). ICU duration was selected over binary ICU admission status for multilinear regression (MLR) as it provided more granular information as a continuous predictor. (refer to Supplementary Material S3; model building explanation)

For the binary logistic regression (BLR) final model, AA-CCI was used to capture a more comprehensive assessment of predictors. The model incorporating AA-CCI demonstrated superior performance (Nagelkerke R^2^ = 80.3%) compared to the model using employment (Nagelkerke R^2^ = 78.3%). Through iterative testing of variable combinations, the final model incorporating AA-CCI, ICU admission, presence of motor impairment, PTA >28 days, admission FIM, intra-rehabilitation medical complications, intra-rehabilitation neurosurgical complications, and discharge destination yielded the highest classification accuracy (80.3%) and was therefore selected as the final BLR model. The level of all statistical tests was *p* < 0.05.

Guidelines from STROBE (www.strobe-statement.org, accessed on 16 May 2025) were followed to strengthen the reporting of the study. (refer Supplementary Materials S4; STROBE checklist).

## 3. Results

Altogether, 289 data sets were analysed from 297 retrieved data sets. A total of 8 datasets were excluded: (age < 21 years (5), major missing data (3). Overall, the median (IQR) RLOS was 28 (21) days, while PRLOS >30 affected 39.8%; these patients’ RLOS was 16 days longer, at median (IQR) 44 (19.5) days. (*p* < 0.001).

Table 1 shows the comparison of baseline demographics and acute TBI characteristics & management by RLOS group. (≤30 days vs. >30 days)

Overall, the cohort consisted of older, Chinese males with predominant TBI etiology of falls, ~60% were employed at TBI onset, and nearly ¾ presented with at least 1 pre-existing comorbidity. Most patients were categorized as mild TBI (mTBI) on emergency-room GCS (GCS 13–15; 58.1%), while 39.1% had moderate to severe TBI (ms-TBI, GCS 8–12).

Comparing patients with RLOS ≤30 days versus >30 days, significant differences were observed in employment status before TBI (*p* = 0.014), AA-CCI (*p* = 0.0215), ICU admission (*p* = 0.0118), and ALOS (*p* < 0.001).

While TBI severity (classified by emergency-room GCS) was mild in 58.1%, 57.1% had PTA > 28 days, (median PTA duration 31 days), indicative of very severe TBI [22]. However, 7.9% (23) patients of our sample were unable to score PTA due to agitation, attention or aphasia thus 266 PTA data sets were available. Demographic factors (age, sex, race), premorbid employment status, and TBI mechanism (falls, road traffic accidents) were not found to significantly correlate with PRLOS > 30. However, higher admission AA-CCI was correlated with lower proportions of PRLOS >30. (Table 2). Admission to the ICU was also significantly correlated with lower proportions of PRLOS, while the effect of neurosurgeries on PRLOS > 30 was not significant. A longer ALOS was also significantly associated with higher proportions of PRLOS > 30. (*p* < 0.001).

Table 2 shows the comparison of rehabilitation characteristics by PRLOS group (≤30 days vs. >30 days).

Univariate analysis of intra-rehabilitation variables correlated with RLOS (days) showed that the following were correlated with a higher proportion of PRLOS > 30: admission motor impairment, lower Ta-FIM, in particular, Ta-FIM ≤ 40, PTA > 28 days, presence of both intra-rehabilitation medical and neurosurgical complications, and discharge to nursing home status. PTA emergence was not significantly correlated with PRLOS > 30. (Table 2) The median FIM gain was Δ27 points without significant differences between shorter RLOS versus PRLOS > 30. FIM efficiency was noted to be significantly lower in PRLOS > 30 compared to those who stayed ≤30 days (0.6 vs. 1.4, *p* < 0.001) (Table 2).

Table 3 shows the total and subset FIM scores upon admission and discharge. There were significant positive gains in all total, motor, and cognitive subset FIM scores from admission to discharge timepoints (*p* < 0.001).

Table 4 shows the results for binary logistic regression for PRLOS > 30 days.

In the final BLR model, 4 variables were significant predictors of PRLOS > 30. These included PTA >28 days (OR 4.01, 95% CI 1.90–8.45, *p* < 0.001), Ta-FIM score ≤40 (OR 4.71, 95% CI 2.32–9.59, *p* < 0.001), presence of neurosurgical complications (OR 4.74, 95% CI 1.28–17.6, *p* = 0.02) and discharge to a non-home destination (OR 2.75. 95% CI 1.12–6.76, *p* = 0.03). The final model was tested on 87 observations and yielded an 80.3% accuracy. Several factors were not found to be significant predictors of PRLOS: AA-CCI, ICU admission, motor impairment, and medical complications (Table 4).

Table 5 shows the results for multilinear regression for mean RLOS (days).

In the final multilinear regression model (Table 5), significant predictors of longer RLOS included PTA duration (β = 0.28, 95% CI 0.17–0.38, *p* < 0.001), admission motor FIM (β = −0.39, 95% CI −0.52 to −0.27, *p* < 0.001), admission cognitive FIM (β = 0.27, 95% CI 0.03–0.52, *p* = 0.03), intra-rehabilitation medical complications (β = 3.38, 95% CI 0.24–6.52, *p* = 0.04), intra-rehabilitation neurosurgical complications (β = 7.58, 95% CI 1.64–13.52, *p* = 0.01), and non-home discharge destination (β = 5.13, 95% CI 0.42–9.84, *p* = 0.03). The moderate predictive power of the multilinear regression model (R^2^ = 0.446) had limited predictive power, indicating that only 44.6% of the variance in RLOS could be explained by our selected variables. This suggests that other unmeasured factors may influence RLOS in TBI rehabilitation. While our model identified significant predictors of RLOS, its modest R ^2^ value of 0.446 limits its utility as a precise predictive tool for individual patient RLOS estimation. Nevertheless, the identified factors provided valuable insights into the key determinants of rehabilitation length of stay in TBI inpatients. (Table 5).

## 4. Discussion

### 4.1. Demographic, Acute TBI Characteristics and Rehabilitation Length of Stay (RLOS)

The purpose of this study was to determine correlates of PRLOS exceeding 30 days and RLOS (days) in an Asian moderate to severe TBI cohort who completed acute inpatient rehabilitation. Data were obtained through retrospective electronic discharge medical charts and the inpatient functional database. We observed that the cohort was representative of older TBI (median age 64 years, 47.8% > 65 years) with a predominance of males (79%). Patients with Chinese ethnicity made up 80% of the sample, in concurrence with the local Chinese racial distribution (75% Chinese) [27]. The leading mechanism of injury was TBI-related low-velocity falls (61.2%), consistent with the main TBI etiologies in older populations (Table 1) [1,2]. Comparing the center’s data over 13 years (2010 to 2023), the mean TBI age at onset of rehabilitation had increased by ~20 years (39.4 years in 2010 vs. 59–60 years in 2023 vs. 60 years in 2025) in the past 15 years [13,18,19,20]. The proportion of TBI-related falls more than doubled during this period (27.5% TBI from falls in 2010 vs. 59.4% in 2023 and 61.2% 2025) [13,18,19,20]. This picture is consistent with rapidly aging TBI epidemiology in Singapore’s superaged status, where 21% of the population will be >65 years of age in 2026 [28].

With regards to RLOS, our median (IQR) RLOS of 28 days (IQR 21) was consistent with previous local studies from 2010–2023, which ranged from 28–30 days, indicating a trend of RLOS stability possibly explained by consistent pre-admission patient selection criteria, TBI rehabilitation programme and discharge criteria, and availability of various post-discharge care schemes, i.e., paid live-in foreign domestic workers, day rehabilitation/senior care centers, and nursing homes [13,18,19,20]. In stark contrast, the global TBI literature indicates a significant downward trend in RLOS over a decade, in spite of older age at TBI onset and worsening functional independence on admission. From 2002 to 2016, the Uniform Data System for Medical Rehabilitation (UDSMR) documented mean RLOS for TBI admitted to medical rehabilitation facilities reduced from 19.0 to 14.5 days alongside an increase in mean patient age and decrease in FIM score from 56.9 to 54.5 on admission, with stability of sex and race [29]. The overall impression was maintenance of discharge FIM despite a shorter RLOS, reflecting a change in TBI injury, possibly explained by the preponderance of older TBI-related falls. Similar trends were also seen for severe TBI admitted to inpatient rehabilitation facilities, where, from 2002 to 2017, mean RLOS decreased significantly from 41.5 to 29.3 days, postulating a possibility of poorer functional outcomes due to shorter time in specialized rehabilitation [29].

Globally, while the influence of racial minorities, FIM discharge attainment, and RLOS (days) cannot be overstated, we did not find significant differences in our cohorts’ RLOS between majority (Chinese) and minority (non-Chinese) races, possibly due non-influence of external payors and the presence of a universal Medisave scheme [11,12,17,29].

The median FIM gain from discharge to admission FIM, was Δ27 (Table 2 and Table 3) (*p* < 0.001) exceeding the Minimal Clinically Important Difference (MCID) of FIM gain Δ25 for TBI inpatient rehabilitation, supporting that a month of acute inpatient rehabilitation after moderate-severe TBI significantly improved functional outcomes in both motor and cognitive FIM subdomains with 83% discharged home [30].

### 4.2. Acute and Rehabilitation Correlates of Prolonged Rehabilitation Length of Stay (PRLOS < 30)

Previous publications have established that older TBI age at onset, lower functional status on admission, presence of comorbidities, and PTA post-TBI led to extended RLOS [31,32,33,34]. Patient comorbidities significantly affect TBI RLOS as hypertension, cardiac conditions, diabetes, and depression are associated with secondary frailty, poorer physical reserve leading to poorer functional outcomes and a longer time to achieve relevant gains [35] We found that severe AA-CCI groups (> 3) paradoxically contributed to lower proportions of PRLOS >30 (22% PRLOS > 30 vs. 27% RLOS < 30 days, *p* = 0.02, Table 1), however, both logistic and linear regression models did not find age nor preinjury employment status nor comorbidities (represented by AA-CCI) to be significant correlates of PRLOS > 30. We postulate that severe AA-CCI groups could be selected out of the rehabilitation programme if they had poor premorbid functional status, and that while the proportions of higher/lower AA-CCI were evenly distributed, lower AACCI categories were likely well controlled and higher AA-CI were under-represented.

While several studies have highlighted a significant impact of TBI severity on functional severity and RLOS, our cohort’s admission GCS mild (13–15) vs. ms-TBI severity did not significantly correlate with RLOS. In older TBI, GCS often underestimates TBI severity as low velocity falls are rarely present with coma or loss of consciousness, but mild TBI in older individuals may result in poor outcomes from a variety of factors. These include delays in diagnosis in older atypical presentations of TBI (e.g., general functional decline), challenges in isolating comorbidities from TBI impairments, cerebral ischemia, early withdrawal of life-sustaining treatment, or impaired mitochondrial function [19,36,37]. Rather, PTA duration during inpatient rehabilitation was significantly associated with PRLOS > 30, as patients with prolonged PTA durations were 4 times more likely to have PRLOS > 30, while every day in PTA was prolonged by 0.2 days of RLOS. (Table 4 and Table 5).

PTA duration is a widely established predictor of outcome for moderate to severe TBI, in terms of discharge functional outcome (FIM), level of care/supervision needed at discharge, global outcomes and late productivity status; with specific thresholds of 4 weeks (good prognosis) and 8 weeks (moderate prognosis) being salient for prognostication for societal outcomes [19,20,29,31,32,33,34]. 22.5% of patients with PTA > 28 days achieved discharge FIM ≥91 compared to 28% of patients with PTA 28 days who achieved FIM ≥91 (*p* < 0.001), confirming the dual negative impact of prolonged PTA periods on functional independence and RLOS, and this has also been confirmed by other authors [29,31,33,34]. We posit that patients with PTA > 28 days have prolonged impaired, continuous working and declarative memories, decreased ability to retain new information affecting functional independence, and some may also have post-traumatic agitation, which is known to contribute to longer RLOS [31,32]. Furthermore, patients in PTA are unable to benefit significantly from speech therapy, and clinical psychological testing to determine cognitive strengths/weaknesses is often only performed post-PTA emergence with adequate arousal levels. However, the ability to emerge from PTA was not associated with mean RLOS or PRLOS > 30, as our discharge criteria did not require emergence from PTA, provided that non-emergent PTA patients had the presence of a trained caregiver upon discharge home.

### 4.3. Regression Analyses of Factors Impacting PRLOS > 30 Days and Mean RLOS (Days)

Overall, 4 intra-rehabilitation factors were identified as significant predictors of PRLOS > 30 (Table 5): PTA duration > 28 days, lower Ta-FIM ≤ 40, non-home discharge, and intra-rehabilitation neurosurgical rather than medical complications were identified. Hence, there was overall agreement with published literature that lower Ta-FIM, longer PTA durations were significant impactors of longer RLOS, Ta-FIM was found to be the most important functional predictor of RLOS, and FIM gains are similar despite RLOS periods [38,39,40,41]. However, FIM efficiency was clearly worse in the longer stay group (0.6 RLOS > 30 days vs. 1.4 RLOS ≤ 30 days, *p* < 0.001) (Table 2), indicating a similar benefit of inpatient rehabilitation but significantly slower rate of gain per day. For patients attaining FIM ≥ 91, only 10% of the sample exceeded 30 days compared to 41% whose RLOS < 30 days (Table 2).

Discharge destination is a well-known predictor of RLOS [39,41,42]. In concurrence, our study identified a higher proportion with PRLOS > 30 for those who were discharged to institutional care, with 5 days added for non-home discharge placement and O.R of 2.75 for PRLOS > 30 for non-home placement. This is likely related to social extensions of RLOS while waiting for nursing home beds or interim placement, after completion of an inpatient TBI rehabilitation programme.

Several large cohorts (1999–2009) had demonstrated good discrimination models with FIM motor and cognitive scores, TBI injury, premorbid education, ALOS, punctate/petechial hemorrhage, and primary payor sources to predict extended RLOS > 67 days [43]. We postulate that in the absence of multimodal imaging data and payor influence, and the older age of our cohort, premorbid education level was not significant, nor was premorbid employment; rather, intra-rehabilitation correlates were predictive.

While acute neurosurgical interventions (e.g., craniectomy or craniotomy vs. conservative treatment) were not associated with prolonged RLOS, both intra-rehabilitation delayed neurosurgical and medical complications were significantly associated with PRLOS > 30. However, only delayed intra-rehabilitation neurosurgical complications significantly predicted prolonged RLOS, increasing the odds of longer rehabilitation stay by 4 times, 7 days of RLOS (*p* = 0.013), while intra-rehabilitation medical complications days increased mean RLOS by 3 days (*p* = 0.036). Neurosurgical complications included hydrocephalus, post-traumatic seizures, new or worsening intracranial collections, sunken-flap syndrome etc. which likely altered consciousness or worsened motor impairment leading to transfer out (ACUR) of the rehabilitation unit to acute neurosurgical wards for neurosurgical consultation, additional time for brain imaging and further surgeries, truncating rehabilitation therapies, thus worsening functional decline and lengthening RLOS, when patients returned to the rehabilitation unit.

The presence of intra-rehabilitation medical complications did not significantly impact PRLOS >30, compared to neurosurgical complications was likely due to a minimal intensity of inpatient rehabilitation occurring during treatment of these complications. This is a common finding amongst studies as complications needing treatment or interrupting rehabilitation due to medical decompensation (i.e., fever, hospital-acquired infections, hemodynamic instability, metabolic derangement worsening or altered mental status) impede the progress of rehabilitation, thereby increasing RLOS [44,45]. Furthermore, the potential impact of hospital-acquired infections in acute ICU patients, e.g., Clostridium difficile infections, has been associated with significant prolongations of ALOS by ~6 to 8 days and increased likelihood of discharge to post-acute rehabilitation or a nursing home [46,47].

With regards to findings from our multilinear regression model (Table 5), the modest explanatory power (44.6%) could possibly be explained by the inability of our limited retrospective data set to account for other relevant clinical, social, or system-level variables, e.g., poor social supports such as living alone, hospital bed occupancy rates, rehabilitation staffing levels. Hence, exploring alternative modelling approaches may improve predictive accuracy for RLOS estimation.

The study’s limitations include: retrospective design, limiting the ability to capture important unmeasured confounders, relatively small sample size from a single centre, proportion of missing data, potential selection biases from pre admission to rehabilitation such as physiatrist screening; where patients could be selected based on perceived potential to benefit from inpatient rehabilitation and patient preference for the rehabilitation centre, lack of data on admission TBI imaging data, cost utilization data and long-term post-discharge outcomes, societal or mortality outcomes. We recognize the limited external generalisability of our findings even in Asia and globally due to data from a single centre and country with predominance of Chinese, and country/centre-specific definitions of prolonged LOS and effects of insurance payors.

## 5. Conclusions

Findings from an older Asian TBI cohort affirm known identified predictors of PRLOS > 30 days; longer PTA duration, lower Ta-FIM < 40/126, and nursing home discharge. Delayed neurosurgical complications during rehabilitation significantly contributed to the longer RLOS of 1 week. These findings suggest that beyond the acute neurotrauma phase, it is important to measure and track PTA duration, promptly recognise and diagnose delayed neurosurgical complications to minimise disruptions to rehabilitation and preserve functional outcome. Targeted intensive locomotor, functional retraining, and advanced discharge planning to facilitate a discharge home could better manage limited rehabilitation resources. A reliable model for the prediction of RLOS (days) remains elusive, thus raising further needs for more robust data from Asian TBI rehabilitation centers.

## Figures and Tables

**Table 1:** Comparison of baseline demographics, acute TBI characteristics by Rehabilitation Length of Stay (RLOS), (n = 289).

Variable	Total (n = 289)	RLOS < = 30 (n = 174)	RLOS >30 (n = 115)	*p*-Value
Age				
Age (years, median (IQR) [95% CI])	64 (28) [61–66]	61.5 (29.8) [58–66]	66 (26.5) [64–70]	0.217 ^a^
Gender, n(%)				
Male	228 (78.9)	139 (48.1)	89 (30.8)	0.525 ^a^
Female	61 (21.1)	35 (12.1)	26 (9)	
Race, n(%)				
Chinese	230 (79.6)	135 (46.7)	95 (32.9)	0.0738 ^a^
Non-Chinese	59 (20.4)	39 (13.5)	20 (6.9)	
AA-CCI, n(%)				
AA-CCI = 0,1, 2	148 (51.2)	96 (33.2)	52 (18)	0.0215 ^a^
AA-CCI ≥ 3	141 (48.8)	78 (27.0)	63 (21.8)	
Employed, n(%), n = 284 *				
Yes	172 (59.5)	112 (38.8)	60 (20.8)	0.014 ^a^
No	112 (38.8)	61 (21.1)	51 (17.6)	
Pre-Injury Comorbidities, n(%)				
Present	206 (71.3)	122 (42.2)	84 (29.1)	0.0554 ^a^
Hypertension	118 (40.8)	72 (24.9)	46 (15.9)	0.502 ^a^
Hyperlipidemia	109 (37.7)	61 (21.1)	48 (16.6)	0.0474 ^a^
Diabetes Mellitus, Type II	68 (23.5)	35 (12.1)	33 (11.4)	0.0821 ^a^
Previous TBI	11 (3.8)	4 (1.4%)	7 (2.4)	0.14 ^a^
Injury Mechanism, n(%)				
Falls	177 (61.2)	108 (37.4)	69 (23.9)	0.171 ^b^
Road Traffic Accident	103 (35.6)	59 (20.4)	44 (15.2)	
Others	9 (3.1)	7 (2.4)	2 (0.7)	
Admission GCS, n(%), n = 281 *				
Mild (13- 15)	168 (58.1)	113 (39.1)	55 (19)	0.0722 ^a^
Moderate and Severe (3–12)	113 (39.1)	56 (19.4)	57 (19.7)	
ICU Admission, n(%), n = 272 *				
Present	179 (61.9)	100 (34.6)	79 (27.3)	0.0118 ^a^
Acute Neurosurgery Type, n(%)				
Craniotomy	64 (22.1)	33 (11.4)	31 (10.7)	0.139 ^a^
Decompressive Craniectomy	31 (10.7)	18 (6.2)	13 (4.5)	0.657 ^a^
ALOS				
Acute LOS (days, median (IQR) [95% CI])	18 (16) [16–19]	16 (12.8) [14–18]	22 (21) [18–26]	<0.001 ^a^

^a^ Wilcoxon Signed Rank Test; ^b^ Kruskal Walis Test; * N = 289, unless otherwise stated; Legend: AA-CCI—Age-Adjusted Charlson Comorbidity Index, TBI—Traumatic Brain Injury, GCS—Glasgow Coma Scale, ICU—Intensive Care Unit, ALOS—Acute Length of Stay.

**Table 2:** Comparison of Rehabilitation Characteristics by Rehabilitation Length of Stay (RLOS), (n = 289).

Variable	Total (n = 289)	RLOS ≤ 30 (n = 174)	RLOS >30 (n = 115)	*p*-Value
LOS Rehab (days, median (IQR) [95% CI])	28 (21) [25–29]	21 (10) [19–22]	44 (19.5) [40–48]	<0.001 ^a^
PTA Duration (days, median (IQR) [95% CI]), n = 266 *	31 (18) [30–32]	28 (10.8) [26–30]	40.5 (31.5) [33–45.5]	<0.001 ^a^
PTA Emergence, n(%), n = 265 *				
Present	214 (74)	136 (47.1)	78 (27)	0.898 ^a^
PTA ≥ 28 Days, n(%), n = 266 *				
Yes	165 (57.1)	79 (27.3)	86 (29.8)	<0.001 ^a^
Motor Impairment, n(%)				
Present	106 (36.7)	47 (16.3)	59 (20.4)	<0.001 ^a^
Medical Complications, n(%)				
Present	149 (51.6)	75 (26)	74 (25.6)	<0.001 ^a^
Neurosurgical Complications, n(%)				
Present	25 (8.7)	6 (2.1)	19 (6.6)	<0.001 ^a^
Discharge Destination, n(%), n = 288 *				
Home	239 (82.7)	159 (55)	80 (27.7)	<0.001 ^a^
Others	49 (17)	14 (4.8)	35 (12.1)	
ACUR, n(%)				
Present	42 (14.5)	16 (5.5)	26 (9%)	0.0015 ^a^
FIM (Admission), n = 287 *				
Total FIM, median (IQR) [95% CI]	57 (37.5) [53–63]	66 (30) [63–71]	37.5 (35.3) [32–45]	<0.001 ^a^
Motor FIM, median (IQR) [95% CI]	39 (29.5) [36–42]	46 (24) [43–49]	25 (24) [22–28]	<0.001 ^a^
Cognitive FIM, median (IQR) [95% CI]	17 (13.5) [16–19]	21 (12) [17–22]	13 (11.8) [10.5–14.5]	<0.001 ^a^
FIM (Discharge), n = 287 *				
Total FIM, median (IQR) [95% CI]	91 (34) [87–95]	100 (28) [95–104]	78.5 (31) [69.5–81.5]	<0.001 ^a^
Motor FIM, median (IQR) [95% CI]	67 (28) [53–81]	74 (23) [71–77]	56 (24.6) [50–58]	<0.001 ^a^
Cognitive FIM, median (IQR) [95% CI]	25 (10) [23–26]	27 (8) [25–28]	22 (11) [20–23]	<0.001 ^a^
Calculated FIM Scores, n = 287 *				
FIM Gain, median (IQR) [95% CI]	27 (29.5) [24–31]	27 (27) [25–31]	24.5 (33.8) [22–34]	0.766 ^a^
FIM Efficiency, median (IQR) [95% CI]	1.1 (1.2) [0.9–1.3]	1.4 (1.2) [1.2–1.6]	0.6 (0.9) [0.5–0.7]	<0.001 ^a^
Ta-FIM ≤ 40, n(%)	85 (29.4)	24 (8.3)	61 (21.1)	<0.001 ^a^
Td-FIM ≥ 91, n(%)	147 (50.9)	117 (40.5)	30 (10.4)	<0.001 ^a^

^a^ Wilcoxon Signed Rank Test; * N = 289, unless otherwise stated.; Legend: LOS—Length of Stay; PTA—Post Traumatic Amnesia; ACUR—Acute Unplanned Return; FIM—Functional Independence Measure; Ta-FIM—Total Admission Functional Independence Measure; Td-FIM—Total Discharge Functional Independence Measure; Formula: FIM Gain = Td-FIM-Ta-FIM, FIM Efficiency = FIM Gain/RLOS (Days).

**Table 3:** Group summary table on admission and discharge FIM (n = 289).

Variable	Admission	Discharge	*p*-Value
Total FIM, median (IQR) [95% CI]	57 (37.5) [53–63]	91 (34) [87–95]	<0.001 ^a^
Motor FIM, median (IQR) [95% CI]	39 (29.5) [36–42]	67 (28) [53–81]	<0.001 ^a^
Cognitive FIM, median (IQR) [95% CI]	17 (13.5) [16–19]	25 (10) [23–26]	<0.001 ^a^

^a^ Wilcoxon Signed Rank Test; Legend: FIM—Functional Independence Measure.

**Table 4:** Binary Logistic Regression of factors associated with PRLOS (>30 Days) (n = 289).

	Exp(β)	95% CI	*p*-Value
AA-CCI (1 = CCI ≥ 3)	1.165	(0.609, 2.230)	0.644
ICU Admission (1 = Present)	1.424	(0.696, 2.914)	0.334
Motor Impairment (1 = Present)	1.707	(0.878, 3.320)	0.115
PTA Duration (1 = PTA Duration > 28 Days)	4.010	(1.903, 8.448)	<0.001
Ta-FIM (1 = Ta-FIM ≤40)	4.711	(2.315, 9.586)	<0.001
Medical Complications (1 = Present)	1.248	(0.651, 2.394)	0.505
Neurosurgical Complications (1 = Present)	4.737	(1.277, 17.57)	0.020
Discharge Destination (1 = Non-Home)	2.745	(1.115, 6.759)	0.028

Legend: AA-CCI—Age Adjusted Charlson Comorbidity Index, ICU—Intensive Care Unit, PTA—Post Traumatic Amnesia, Ta-FIM—Total Admission Functional Independence Measure, PRLOS—Prolonged Rehabilitation Length of Stay, exp (β)—exponential of beta coefficient.

**Table 5:** Multilinear Regression for factors associated with RLOS (Days) (n = 289).

	β	95% CI	*p*-Value
Employed Before TBI (1 = Yes)	0.525	(−2.799, 3.849)	0.757
ICU Duration (days)	−0.298	(−0.701, 0.104)	0.148
ALOS (days)	0.138	(−0.014, 0.291)	0.077
Motor Impairment (1 = Present)	1.863	(−1.546, 5.270)	0.285
PTA Duration (days)	0.276	(0.173, 0.380)	<0.001
Admission FIM (Motor)	−0.394	(−0.515, −0.273)	<0.001
Admission FIM (Cognitive)	0.273	(0.026, 0.519)	0.031
Medical Complications (1 = Present)	3.380	(0.243, 6.518)	0.036
Neurosurgical Complications (1 = Present)	7.579	(1.637, 13.52)	0.013
Discharge Destination (1 = Non-Home)	5.128	(0.418, 9.838)	0.034

Legend: TBI—Traumatic Brain Injury, ICU—Intensive Care Unit, ALOS—Acute Length of Stay, PTA—Post Traumatic Amnesia, FIM—Functional Independence Measure, β—Beta Coefficient, RLOS—Rehabilitation Length of Stay.

## Data Availability

The data presented in this study are available on request from the corresponding author due to ethical reasons.

## Supplementary Materials

The following supporting information can be downloaded at: https://www.mdpi.com/article/10.3390/life15071136/s1, S1: Ethics Approval. S2: List of Variables (LOV); S3: multilinear regression model explanation. S4: STROBE Statement.
