# Supplementary material for: Correlates of Rehabilitation Length of Stay in Asian Traumatic Brain Injury Inpatients in a Superaged Country: A Retrospective Cohort Study
Source: Life (Basel). 2025 Jul 18;15(7):1136. doi: 10.3390/life15071136 (PMC12297994; doi:10.3390/life15071136)
Supplement: Supplementary file 1 [file life-15-01136-s001.zip › Data supplement file S3.pdf]

Data supplement file 3:

Explanation of multilinear regression model

MODEL A: Base Model

Our initial base model ($R^2 = 0.4103$), after variable selection had 9 variables in total. The variables used are, ICU days, ALOS, presence of motor impairment, presence of neurosurgical complications, PTA duration, discharge destination, medical complications, Ta-FIM (total admission FIM) Score and CCI. Through iterative model development, we explored several variable combinations.

MODEL B

We first compared continuous versus binary PTA duration (>28 days). The continuous specification (days) demonstrated better model performance ($R^2 = 0.4103$) compared to the binary categorization ($R^2 = 0.3807$) and was retained in subsequent models.

MODEL C, D(a),D(b), E

After retaining PTA duration as a continuous variable, the next iteration tested CCI as a binary variable (AA CCI) instead of its continuous form. This modification resulted in a slight improvement in model performance ($R^2 = 0.4125$ vs $R^2 = 0.4103$). Subsequently, AA CCI was removed and replaced with age as a continuous variable, which led to a slightly lower model performance ($R^2 = 0.4110$). The addition of employment status further improved model performance ($R^2 = 0.4218$), suggesting its value as a socioeconomic predictor of rehabilitation length of stay. ACUR (transfer out during rehabilitation stay) demonstrated significant collinearity with both neurosurgical complications ($\chi^2 = 41.63$, p = <0.001) through Chi-square testing. Due to significant collinearity between ACUR and neurosurgical complications ($\chi^2 = 41.63$, $p < 0.001$), only one variable was retained. The model incorporating ACUR showed slightly lower performance ($R^2 = 0.4129$) and therefore neurosurgical complications was retained for subsequent analyses.

MODEL F: Final Model

The initial model specification included Ta-FIM score as a single measure of functional independence. However, when the components were added separately in the final model, we found distinct relationships: admission motor FIM demonstrated a significant negative association with rehabilitation length of stay ($\beta = -0.394$, $p < 0.001$), while admission cognitive FIM showed a significant positive association ($\beta = 0.273$, $p = 0.031$). The separation of FIM components improved model performance ($R^2 = 0.4457$). (Table 5)

MODEL G

Log transformation of the dependent variable (RLOS) was explored and although it improved model performance ($R^2 = 0.4732$), it was not selected as the final model due to reduced clinical interpretability. The untransformed model was retained to maintain direct interpretation of the relationships between predictors and RLOS in days.
