# Supplementary material for: Correlates of Rehabilitation Length of Stay in Asian Traumatic Brain Injury Inpatients in a Superaged Country: A Retrospective Cohort Study
Source: Life (Basel). 2025 Jul 18;15(7):1136. doi: 10.3390/life15071136 (PMC12297994; doi:10.3390/life15071136)
Supplement: Supplementary file 1 [file life-15-01136-s001.zip › Data supplement S4_STROBE_TBIRLOS_CHUAKSG.pdf]

STROBE Statement—checklist of items that should be included in reports of observational studies

Title: Correlates of rehabilitation length of stay in Asian traumatic brain injury inpatients in a superaged country

	Item No	Recommendation
**Title and abstract**	1	(*a*) Indicate the study's design with a commonly used term in the title or the abstract: >>Retrospective cohort study (title page and abstract, page 1,2)
		(*b*) Provide in the abstract an informative and balanced summary of what was done and what was found >>Abstract
**Introduction**		
Background/rationale	2	Explain the scientific background and rationale for the investigation being reported >>Introduction para 1
Objectives	3	State specific objectives, including any prespecified hypotheses >>Introduction: para 4
**Methods**		
Study design	4	Present key elements of study design early in the paper >>Materials and methods (A)
Setting	5	Describe the setting, locations, and relevant dates, including periods of recruitment, exposure, follow-up, and data collection >>Materials and methods: section A,B,C
Participants	6	*(a)* *Cohort study*—Give the eligibility criteria, and the sources and methods of selection of participants. Describe methods of follow-up (NA) *>>materials and methods: section C* *Case-control study*—Give the eligibility criteria, and the sources and methods of case ascertainment and control selection. Give the rationale for the choice of cases and controls: NA *Cross-sectional study*—Give the eligibility criteria, and the sources and methods of selection of participants: NA
		(*b*) *Cohort study*—For matched studies, give matching criteria and number of exposed and unexposed; NA *Case-control study*—For matched studies, give matching criteria and the number of controls per case; NA
Variables	7	Clearly define all outcomes, exposures, predictors, potential confounders, and effect modifiers. Give diagnostic criteria, if applicable >>materials and methods: section C,E
Data sources/ measurement	8*	For each variable of interest, give sources of data and details of methods of assessment (measurement). Describe comparability of assessment methods if there is more than one group (NA) >>materials and methods: section F: statistics
Bias	9	Describe any efforts to address potential sources of bias >> section F; statistics and study limitations
Study size	10	Explain how the study size was arrived at NA as non-interventional retrospective study

Quantitative variables	11	Explain how quantitative variables were handled in the analyses. If applicable, describe which groupings were chosen and why<br>>>section F: statistics
Statistical methods	12	*(a)* Describe all statistical methods, including those used to control for confounding<br>>>Statistics Analysis: section F
		(b) Describe any methods used to examine subgroups and interactions<br>>>Results: Table 1<br>>> Results: Table 2
		(b) Explain how missing data were addressed:<br>>>table legends (table 1-5) explain each variable which has missing data and number.<br>No data imputation was done study as non-interventional and as only 2 time points from admission to discharge were collected, imputation was not appropriate.
		(*d*) *Cohort study*—If applicable, explain how loss to follow-up was addressed: NA as there was no follow up phase as study was non-interventional and this was not hypotheses driven.<br>*Case-control study*—If applicable, explain how matching of cases and controls was addressed: NA<br>*Cross-sectional study*—If applicable, describe analytical methods taking account of sampling strategy: NA
		(*e*) Describe any sensitivity analyses<br>>>refer to section F: statistics<br>>>refer to data supplement attachment #3 on regression model building

Continued on next page

Results

Participants	13*	(a) Report numbers of individuals at each stage of study—e.g. numbers potentially eligible, examined for eligibility, confirmed eligible, included in the study, completing follow-up, and analysed<br>>>Results: first paragraph
		(b) Give reasons for non-participation at each stage: NA
		(c) Consider use of a flow diagram: NA as a retrospective study design and there was no intervention/follow up phase.
Descriptive data	14*	(a) Give characteristics of study participants (eg demographic, clinical, social) and information on exposures and potential confounders<br>>>Results: table 1 and 2 : characteristics of participants<br>Information on potential confounders<br>>> Sensitivity Analysis: section F statistics
		(b) Indicate number of participants with missing data for each variable of interest:<br>>> indicated in tables 1,2 3 under table legends and next to variable with missing data in main table
		(c) *Cohort study*—Summarise follow-up time (eg, average and total amount)<br>>>Results: section E
Outcome data	15*	*Cohort study*—Report numbers of outcome events or summary measures over time<br>>>Results: table 1,2,3
		Case-control study—Report numbers in each exposure category, or summary measures of Exposure: NA
		Cross-sectional study—Report numbers of outcome events or summary measures: NA

Main results	16	(*a*) Give unadjusted estimates and, if applicable, confounder-adjusted estimates and their precision (eg, 95% confidence interval). Make clear which confounders were adjusted for and why they were included<br>>>section F: statistics, tables 4,5 (refer to 95% CI)
		(c) Report category boundaries when continuous variables were categorized<br>>> Results: Table 1 and Table 2
		(*c*) If relevant, consider translating estimates of relative risk into absolute risk for a meaningful time period<br>>>NA
Other analyses	17	Report other analyses done—eg analyses of subgroups and interactions, and sensitivity Analyses<br>>>table 4: Binary logistic regression<br>>>table 5: Multilinear regression<br>>>sensitivity analysis (section F: statistics)
**Discussion**		
Key results	18	Summarise key results with reference to study objectives<br>>>Discussion: section A
Limitations	19	Discuss limitations of the study, taking into account sources of potential bias or imprecision.<br>>> Discussion: section D: study limitations and comment on generalizability<br>Discuss both direction and magnitude of any potential bias<br>>> Table 5 and its following paragraph
Interpretation	20	Give a cautious overall interpretation of results considering objectives, limitations, multiplicity of analyses, results from similar studies, and other relevant evidence<br>>>Discussion: section B and C
Generalizability	21	Discuss the generalizability (external validity) of the study results<br>>>Discussion section D: study limitations
**Other information**		
Funding	22	Give the source of funding and the role of the funders for the present study and, if applicable, for the original study on which the present article is based<br>>> NA as study was conducted without funding (refer to notes section after references)

*Give information separately for cases and controls in case-control studies and, if applicable, for exposed and unexposed groups in cohort and cross-sectional studies.

Note: An Explanation and Elaboration article discusses each checklist item and gives methodological background and published examples of transparent reporting. The STROBE checklist is best used in conjunction with this article (freely available on the Web sites of PLoS Medicine at http://www.plosmedicine.org/, Annals of Internal Medicine at http://www.annals.org/, and Epidemiology at http://www.epidem.com/). Information on the STROBE Initiative is available at www.strobe-statement.org.
