# Supplementary material for: Correlates of Rehabilitation Length of Stay in Asian Traumatic Brain Injury Inpatients in a Superaged Country: A Retrospective Cohort Study
Source: Life (Basel). 2025 Jul 18;15(7):1136. doi: 10.3390/life15071136 (PMC12297994; doi:10.3390/life15071136)
Supplement: Supplementary file 1 [file life-15-01136-s001.zip › Data supplement file S2_LOV_2DSRB2023_00873.pdf]

For DSRB Application / PorT
**VARIABLE**
**Demographic and socio-economics**
Age as at first TR transfer
Gender
Race
Nationality
Marital Status
Living With
Education
Employment
Caregiver Stress
Strained Relationships
Lack of Social Support
Presence of Behavioural Problems
Presence of Psychiatric Problems
Presence of Confusion
Postal Code
Housing Type
Rental Flat (Yes/No)
**Length of Stays and transfers**
Admission Datetime (in MM-YYYY)
Discharge Datetime (in MM-YYYY)
Time of 1st transfer into TR (in MM-YYYY)
Length of Stay (Overall)
Length of Stay (Acute)
Length of Stay (Sub-Acute, Non-TR)
Length of Stay (Sub-Acute, TR) (i.e. RLOS)
Length of Stay (ICU)
No. of transfers from TR to Acute
No. of transfers from Acute to TR (including first)
**Admission Characteristics**
Admission Type (EM/EL/SD/etc)
Patient Class on Admission
**Discharge Characteristics**
Patient Class on Discharge
Discharge Destination<br>- CH, NH, Died, Absconded/AMA, Transfer to Other Hospitals, Home, Others
Carer on Discharge
**Discharge Diagnoses**
Discharge Primary Diagnosis
Segment Grouping (OTR, STR, SCI, etc)
Comorbidity Indicators (17 Charlson Categories)
Charlson Comorbidiity Index
**Infections**
MRSA
VRE
CPE
**Therapy**
PT

OT
ST
**Diagnostics**
Telemetry
Echocardiography
Wait Time for Radiology Procedures
**Referrals**
Referrals to NH
Referrals to CH
Referrals to MSW
Referrals to IDCT/IDCP
**Surgeries**
Surgical Datetime (in MM-YYYY)
Surgical Code
Surgical Description
Operation Table Code
**Vital Signs**
Glasgow Coma Scale (GCS)
**Complications**
Any infections
Bleeding diaresis
Acute kidney injury
Venous thrombotic events
Dysphagia
NGT or PEG
Decubitus
**Common intra rehab characteristics**
ICH<br>- Mechanism ICH: hypt,CAA, others<br>- Treatment: EVD, craniotomy, craniectomy
WFNS for SAH
Ischaemic Stroke<br>- NIHSS, mechanism (LVO, ICAD,CE,EDUS others)<br>- RTPA, EVT, Conservative
Spinal cord injury<br>- ASIA score<br>- Neurological level of injury (NLI)
Orthopedics<br>- Fractures<br>- Amputees<br>- K score<br>- AMPPRO vs AMPNOPRO
**Functional, complications, impairment characteristics**
iADL and bADL on admission and discharge
MBI on admission and discharge
FIM on admission and discharge<br>- Total motor<br>- Total cog

FMA<br>- Total prox<br>- Total distal
HADS
EQ-5D (5 qns with 5 options) (ordinal)
10metres walk test on admission and discharge
AUSTOMS
Functional Oral Intake Scale (FOIS)
6mins walk test on admission and discharge
FMS on admission and discharge
SAFE and TWIST scores (if applicable)
Menu and track
Trunk Impairment Scale (TIS)
ICH score (for TBI)
Duration of pTA (for TBI)
BBS (if applicable)
Aphasia
MOCA
**Lab or pharmacological / laboratory characteristics**
Anemia – Serum hemoglobin
Hypoglycemia (POCT and serum glucose)
Serum albumin
Anticoagulation use - aspirin, clopidogrel, NOAC (eg apixaban),<br>SC clexane, IV clexane, warfarin
C-reactive Protein (CRP)
Full Blood Count (FBC)
Liver Function
Procalcitonin
Blood Culture
Urine Culture
**Hospital Bill**
Bill Size
