# Supplementary material for: Correlates of Rehabilitation Length of Stay in Asian Traumatic Brain Injury Inpatients in a Superaged Country: A Retrospective Cohort Study
Source: Life (Basel). 2025 Jul 18;15(7):1136. doi: 10.3390/life15071136 (PMC12297994; doi:10.3390/life15071136)
Supplement: Supplementary file 1 [file Data supplementary file_Ethics Approval S1.pdf › Data supplementary file_Ethics Approval S1.pdf]

3 Fusionopolis Link
#03-08 Nexus@one-north
Singapore 138543
Tel: 6496 6600 Fax:6486 6870
www.nhg.com.sg
RCB No. 200002150H

NHG DSRB Ref: **2023/00873**

09 May 2024

Dr Kenneth Ngoh Jun Hong
Department of Gastroenterology & Hepatology
Tan Tock Seng Hospital

Dear Dr Ngoh

**NHG DOMAIN SPECIFIC REVIEW BOARD (DSRB) APPROVAL**

**STUDY TITLE: Machine Learning Predictive Analysis of Key Factors Influencing Rehabilitation Length of Stay (RLOS) and Direct Hospitalization Costs for Neurological Inpatient Rehabilitation at Tertiary Care Hospital**

We are pleased to inform you that the NHG Domain Specific Review Board has approved the application as titled above to be conducted in **Tan Tock Seng Hospital**.

The approval period is from **09 May 2024** to **08 May 2025**. The NHG DSRB reference number for this study is **2023/00873**. Please use this reference number for all future correspondence.

In accordance with the NHG Data Access & Sharing Policy, NHG investigators and study team members who require access to data that is not collected or created directly by the research team from research subjects or research materials (e.g., through interviews, surveys or experiments) should consult the relevant Data Exchange Office (DXO) to ascertain if a data access request should be raised. The NHG Institutional and Cluster DXO's details can be found at https://mynhg.nhg.com.sg/clst/nhgdxo/Pages/default.aspx.

The documents reviewed are:

a) NHG DSRB Application Form: **Version No. 1**

b) List of Variables: **Version 2 dated 02 May 2024**

The NHG DSRB has approved your request for waiver of informed consent.

Continued approval is conditional upon your compliance with the following requirements:

1. Translated Informed Consent Forms (fully translated and short consent forms) and other translated documents are not required to be submitted to DSRB. It is the responsibility of the Principal Investigator to ensure that the translations for any document are an accurate reflection of the original approved content and to maintain the certification/documentation of the translations.

2. No deviation from or changes to the study should be implemented without documented approval from the NHG DSRB, except where necessary to eliminate apparent immediate hazard(s) to the study subjects.

3. Any deviation from or changes to the study to eliminate an immediate hazard should be promptly reported to the NHG DSRB within seven calendar days.

4. Principal Investigators and study team members are responsible for adhering to institutional guidelines, where applicable.

5. Please note that for studies requiring CTA/CTN/CTC, apart from the approval from NHG DSRB, no deviation from, or changes of the Research Protocol and Informed Consent Form should be implemented without documented approval from the Health Sciences Authority unless otherwise advised by the Health Sciences Authority.

6. Please submit the following to the NHG DSRB:

   a. All Unanticipated Problems Involving Risk To Subjects Or Others (UPIRTSOs) must be reported to the NHG DSRB. For more than minimal risk studies, all problems involving local deaths **must be reported as soon as possible**, but not later than 7 calendar days after first knowledge by the Investigator, regardless of the causality and expectedness of the death event, and any additional relevant information about the death should be reported within 8 calendar days of making the initial report. For no more than minimal risk studies, only problems involving local deaths that are related or possibly related to the study **must be reported as soon as possible**, but not later than 7 calendar days after first knowledge by the Investigator, and any additional relevant information about the death should be reported within 8 calendar days of making the initial report. For problems which are life threatening, it **must be reported as soon as possible**, but not later than 7 calendar days after first knowledge by the investigator, and any additional relevant information about the problems should be reported within 8 calendar days of making the initial report. All other problems that fulfil the UPIRTSOs reporting criteria **must be reported as soon as possible** but not later than 15 calendar days after first knowledge by the Investigator.

b. Report(s) on any new information that may adversely affect the safety of the subject or the conduct of the study.

c. NHG DSRB Study Status Report Form – this is to be submitted 4 to 6 weeks prior to expiry of the approval period. The study cannot continue beyond **08 May 2025** until approval is renewed by the NHG DSRB.

d. Study completion – this is to be submitted using the NHG DSRB Study Status Report Form within 4 to 6 weeks of study completion.

With the enactment of the Human Biomedical Research Act, Health Products Act, Medicines Act and their subsidiary legislations, Principal Investigators are reminded to ensure that their research complies with the regulatory requirements stipulated in the applicable Acts. Contraventions under any of these Acts are criminal offences and would result in fines or imprisonment or both, subject to the nature of the offence.

Established since May 2006, the NHG Research Quality Management (RQM) Programme seeks to promote the responsible conduct of research in a research culture with high ethical standards, identify potential systemic weaknesses and make recommendations for continual improvement. Hence, this research study may be randomly selected for a review by the Research Quality Management (RQM) team. For more information, please visit www.research.nhg.com.sg.

The NHG DSRB operates in accordance to the ICH GCP and all applicable laws and regulations.

Yours Sincerely

A/Prof Koh Kwong Fah
Deputy Chairman
NHG Domain Specific Review Board D

Cc: Institutional Representative, TTSH
c/o Clinical Research & Innovation Office, TTSH
Departmental Representative of Gastroenterology & Hepatology, TTSH

*(This is an electronic-generated letter. No signature is required.)*
